# Investigating phase separation properties of chromatin-associated proteins using gradient elution of 1,6-hexanediol

**DOI:** 10.1186/s12864-023-09600-1

**Published:** 2023-08-28

**Authors:** Peiyu Zhu, Chao Hou, Manlin Liu, Taoyu Chen, Tingting Li, Likun Wang

**Affiliations:** 1 Department of Biomedical Informatics, School of Basic Medical Sciences, Peking University Health Science Center, Beijing, 100191 China; 2 The MOE Key Laboratory of Cell Proliferation and Differentiation, School of Life Sciences, Peking University, Beijing, 100871 China; 3 Key Laboratory for Neuroscience, Ministry of Education/National Health Commission of China, Peking University, Beijing, 100191 China; 4 Institute of Systems Biomedicine, Department of Pathology, School of Basic Medical Sciences, Peking University Health Science Center, Beijing, 100191 China

**Keywords:** Liquid–liquid phase separation, Chromatin-associated proteins, 1,6-hexanediol, High-throughput experimental methods

## Abstract

**Background:**

Chromatin-associated phase separation proteins establish various biomolecular condensates via liquid–liquid phase separation (LLPS), which regulates vital biological processes spatially and temporally. However, the widely used methods to characterize phase separation proteins are still based on low-throughput experiments, which consume time and could not be used to explore protein LLPS properties in bulk.

**Results:**

By combining gradient 1,6-hexanediol (1,6-HD) elution and quantitative proteomics, we developed chromatin enriching hexanediol separation coupled with liquid chromatography-mass spectrometry (CHS-MS) to explore the LLPS properties of different chromatin-associated proteins (CAPs). First, we found that CAPs were enriched more effectively in the 1,6-HD treatment group than in the isotonic solution treatment group. Further analysis showed that the 1,6-HD treatment group could effectively enrich CAPs prone to LLPS. Finally, we compared the representative proteins eluted by different gradients of 1,6-HD and found that the representative proteins of the 2% 1,6-HD treatment group had the highest percentage of IDRs and LCDs, whereas the 10% 1,6-HD treatment group had the opposite trend.

**Conclusion:**

This study provides a convenient high-throughput experimental method called CHS-MS. This method can efficiently enrich proteins prone to LLPS and can be extended to explore LLPS properties of CAPs in different biological systems.

**Supplementary Information:**

The online version contains supplementary material available at 10.1186/s12864-023-09600-1.

## Background

The formation of membraneless organelles by liquid–liquid phase separation (LLPS) is a novel biological concept with fast-growing attention [1]. Multiple chromatin-associated proteins (CAPs) forming nuclear condensates through LLPS involve in regulating vital biological processes. For example, heterochromatin protein 1α (HP1α) is critical in forming heterochromatin domains in the nucleus [2, 3]. In addition, condensate formation of RNA Pol II and several transcription factors (TFs) and transcription cofactors (TCs) are critical for gene regulation [4–8]. Therefore, investigating LLPS properties of CAPs assists in elucidating phase separation processes regulating chromatin-associated biological processes. Several in vivo and in vitro low-throughput experimental methods are available to characterize LLPS proteins, including immunofluorescence, droplet roundness/fusion, and fluorescence recovery after photobleaching [9]. However, these methods are time-consuming, and cannot identify LLPS proteins in large scale. In addition, the formation of biomolecular condensate is related to the microenvironment that surrounds the condensate. However, these methods neglect physiological conditions. Given the complexity and difficulty of low-throughput experimental identification of LLPS proteins under physiological conditions, an efficient high-throughput experiment is urgently required to investigate the LLPS properties of CAPs.

1,6-Hexanediol (1,6-HD) is an aliphatic alcohol that interferes with hydrophobic interactions and is commonly used in vivo and in vitro to disassemble LLPS-dependent biomolecular condensates [10, 11]. 1,6-HD interferes not only with hydrophobic interactions between proteins but also with hydrophobic interactions between proteins and nucleic acids, which are required for the formation of LLPS-dependent biomolecular condensates [12]. A previous study has shown that after treating MCF7 cells with 1,6-HD, different proteins exhibited varying degrees of decrease in ChIP-seq signals [6]. This finding suggested different proteins vary in 1,6-HD sensitivity, resulting in different chromatin binding abilities of proteins. This protein-specific sensitivity to 1,6-HD provides a valuable opportunity to investigate the LLPS properties of CAPs in bulk under physiological conditions. Previously, we reported a new high-throughput experimental method called Hi-MS combining 10% 1,6-HD treatment, and found that CAPs have varying 1,6-HD sensitivities, thus reflecting their abilities to bind DNA [13]. However, there are a few limitations regarding Hi-MS: 1) it is complex and time consuming and 2) it only uses one single-concentration for 1,6-HD treatment, although we noticed that CAPs showed different sensitivities to different concentrations of 1,6-HD [13]. Considering that several studies have applied different concentrations of 1,6-HD to disrupt a variety of biomolecular condensates [7, 14, 15], we believe that applying different concentrations of 1,6-HD can assist in better understanding of the LLPS properties of CAPs. Therefore, we need to develop convenient and less time-consuming high-throughput experimental methods to investigate the sensitivity of proteins to different concentrations of 1,6-HD treatment.

In this work, we developed a high-throughput experimental method called chromatin enriching hexanediol separation coupled with liquid chromatography mass spectrometry (CHS-MS) using gradient 1,6-HD elution. CHS-MS could effectively enrich CAPs prone to LLPS. Combining gradient 1,6-HD treatment, we explored the sensitivity of CAPs to 1,6-HD with different concentrations and further examined the physicochemical characteristics of these CAPs.

## Results

### CHS-MS effectively captures chromatin-associated proteins

**Fig. 1:**
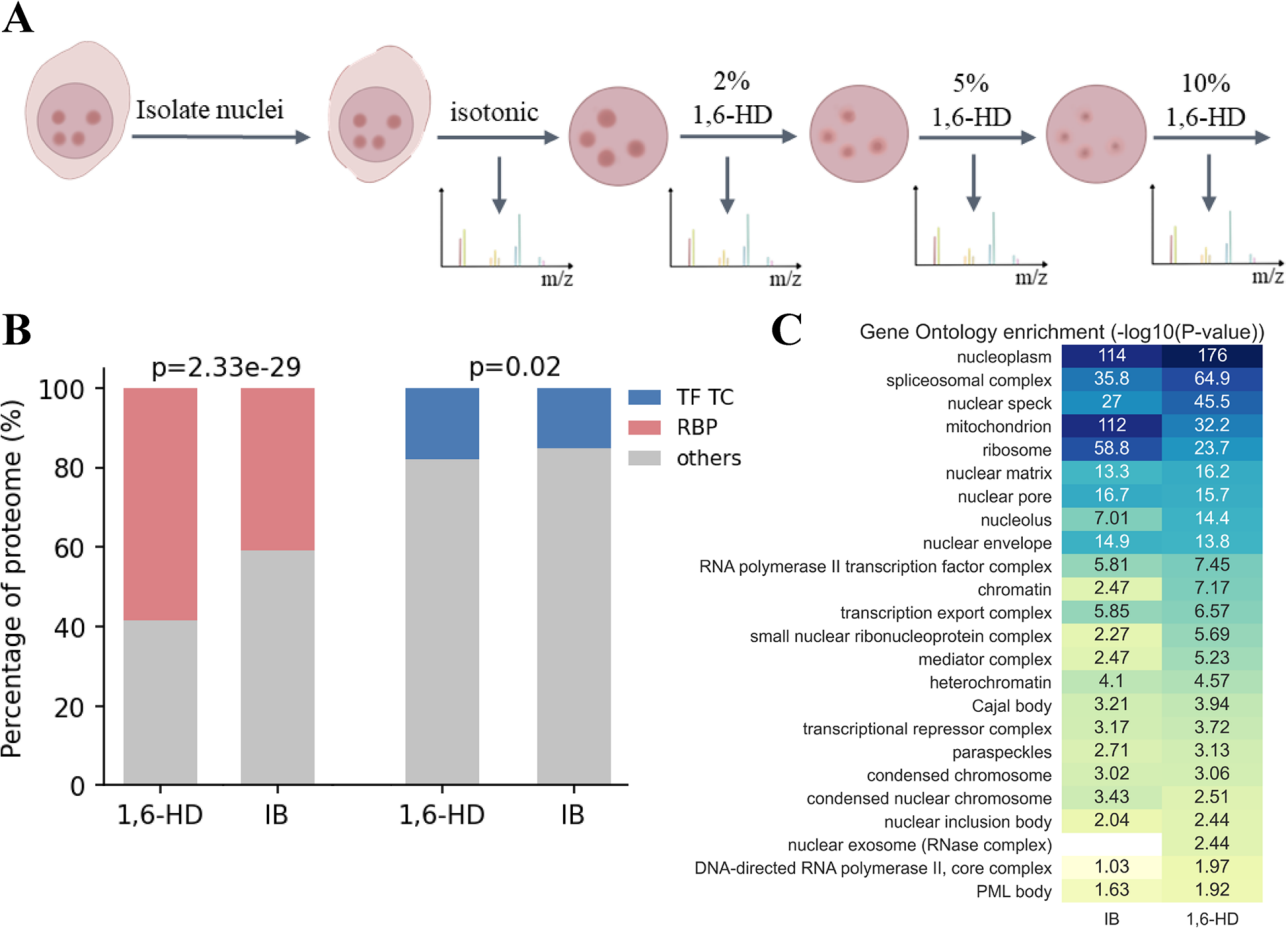
CHS-MS effectively enriches chromatin-associated proteins. **A** Schematic of CHS-MS. **B** Different protein types detected in CHS-MS. *P*-value was calculated using chi-square test. TF, transcription factor; TC, transcription cofactor; RBP, RNA binding protein. **C** Gene Ontology enrichment analysis of proteins. -Log10(*P*-value) was noted in the corresponding cell

In order to explore the LLPS properties of CAPs, we developed a method to capture CAPs (Fig. 1A). To avoid the interference of background proteins, we treated K562 cells with isotonic buffer (IB) to remove proteins in the nucleoplasm and cytoplasm. Subsequently, to investigate the sensitivity of proteins to different concentrations of 1,6-HD, we performed gradient elution to enrich proteins eluted under different conditions (2%, 5%, and 10% 1,6-HD). Finally, we obtained the abundance of proteins in each treatment group with label-free quantitative mass spectrometry (MS). We named this method chromatin enriching hexanediol separation coupled with liquid chromatography mass spectrometry (CHS-MS) (Fig. 1A).

Using this approach, we captured 2,522 (IB), 1,613 (2% 1,6-HD), 1,544 (5% 1,6-HD) and 1,638 proteins (10% 1,6-HD) respectively (Additional file 1: Figure S1A, Additional file 2: Table S1). The 1,6-HD treatment group and the IB treatment group had 1,621 overlapping proteins (Additional file 1: Figure S1B). We analysed the types of proteins enriched in IB and 1,6-HD treatment, and found that 1,6-HD treatment enriched more TFs, TCs, and RNA binding proteins (RBPs) (Fig. 1B). In addition, we performed Gene Ontology (GO) enrichment analysis of the captured proteins in the 1,6-HD and IB treatment groups. As shown in Fig. 1C and Additional file 3: Table S2, GO terms localized in the nucleus, e.g. nucleolus, spliceosomal complex, nuclear speckle, and chromatin, were more enriched in 1,6-HD treatment group, while GO terms localized in the cytoplasm, e.g. ribosome and mitochondrion, were more enriched in IB treatment group.

Taken together, these results suggested that our experimental procedure could effectively enrich CAPs.

### 1,6-HD-eluted proteins are prone to LLPS

**Fig. 2:**
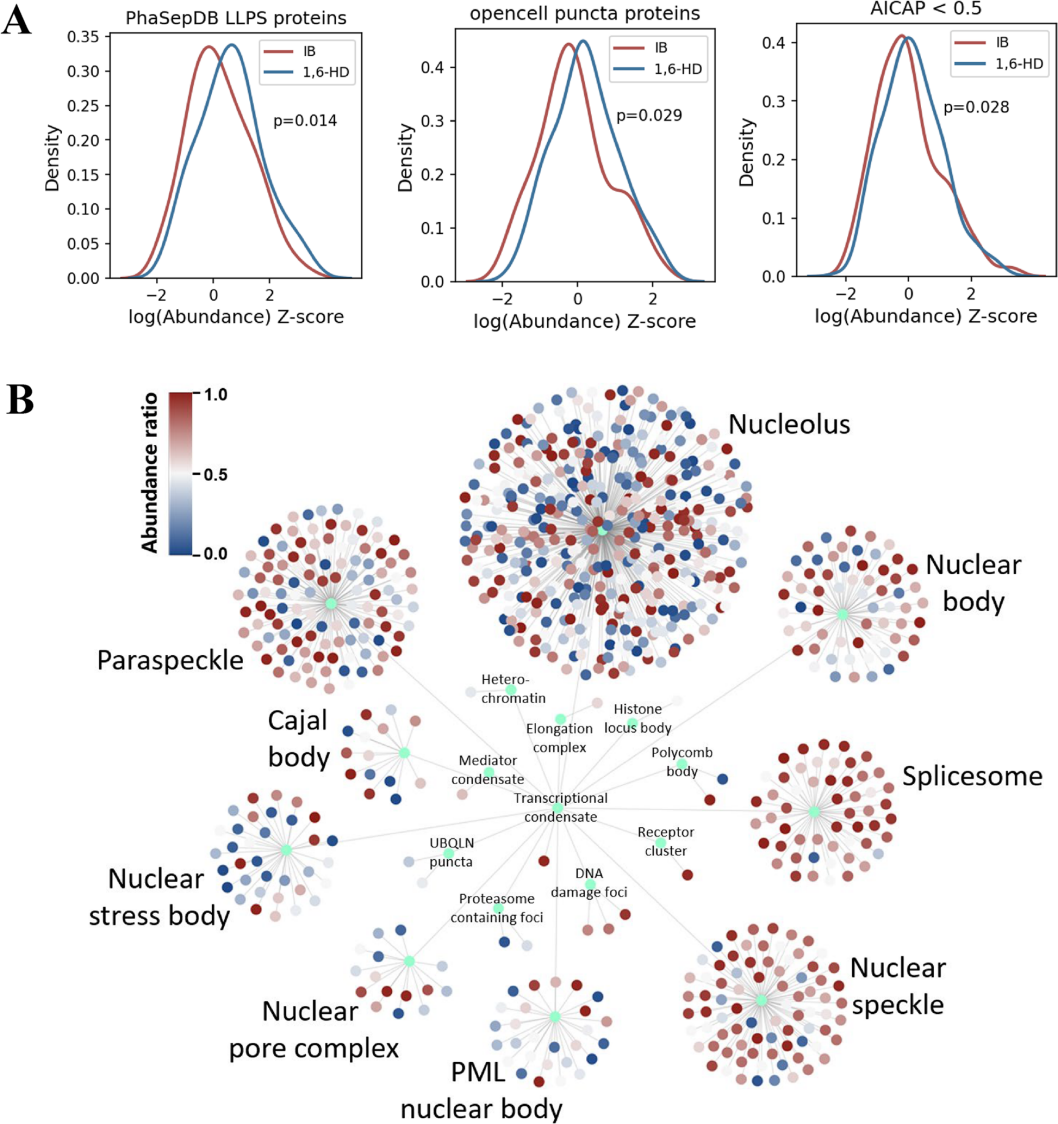
Enrichment of proteins in biomolecular condensates. **A** Distribution of normalized log2(abundance) for LLPS proteins, puncta proteins, and AICAP < 0.5 proteins. The log2(abundance) of proteins was normalized to Z-scores for each treatment. *P*-value was calculated using independent samples t test. LLPS, liquid–liquid phase separation. AICAP, anti-1,6-HD index of chromatin-associated protein. **B** Distribution of abundance ratio of proteins in different condensates. Abundance ratio was calculated from the ratio of protein abundance captured in the 1,6-HD treatment group to the total protein abundance captured in the IB and 1,6-HD treatment groups. 1,6-HD, 1,6-hexanediol

To elucidate the abundance distribution of known phase separation proteins in different treatment groups, we analysed 272 known phase separation proteins from PhaSepDB [16] and found that the 1,6-HD treatment group enriched greater abundance of these known phase separation proteins than the IB treatment group (Fig. 2A). In addition, OpenCell [17] provided extensive protein imaging data. We found that the 1,6-HD treatment group enriched higher abundance of nuclear puncta proteins than the IB treatment group (Fig. 2A). Hi-MS provided an anti-1,6-HD index of chromatin-associated proteins (AICAP) [13]. We found that 1,6-HD treatment group enriched higher abundance of AICAP < 0.5 proteins than the IB treatment group (Fig. 2A). Next, we also investigated the enrichment of various biomolecular condensates-associated proteins from PhaSepDB in different treatment groups. As shown in Fig. 2B and Additional file 4: Figure S2, the 1,6-HD treatment group enriched higher abundance of proteins related to chromatin-associated condensates (nuclear speckle, spliceosome, paraspeckle and nuclear body), whereas the IB treatment group enriched higher abundance of proteins related to biomolecular condensates closely associated with cytoplasm (nucleolus, nuclear pore complex, and nuclear stress body) [18].

**Fig. 3:**
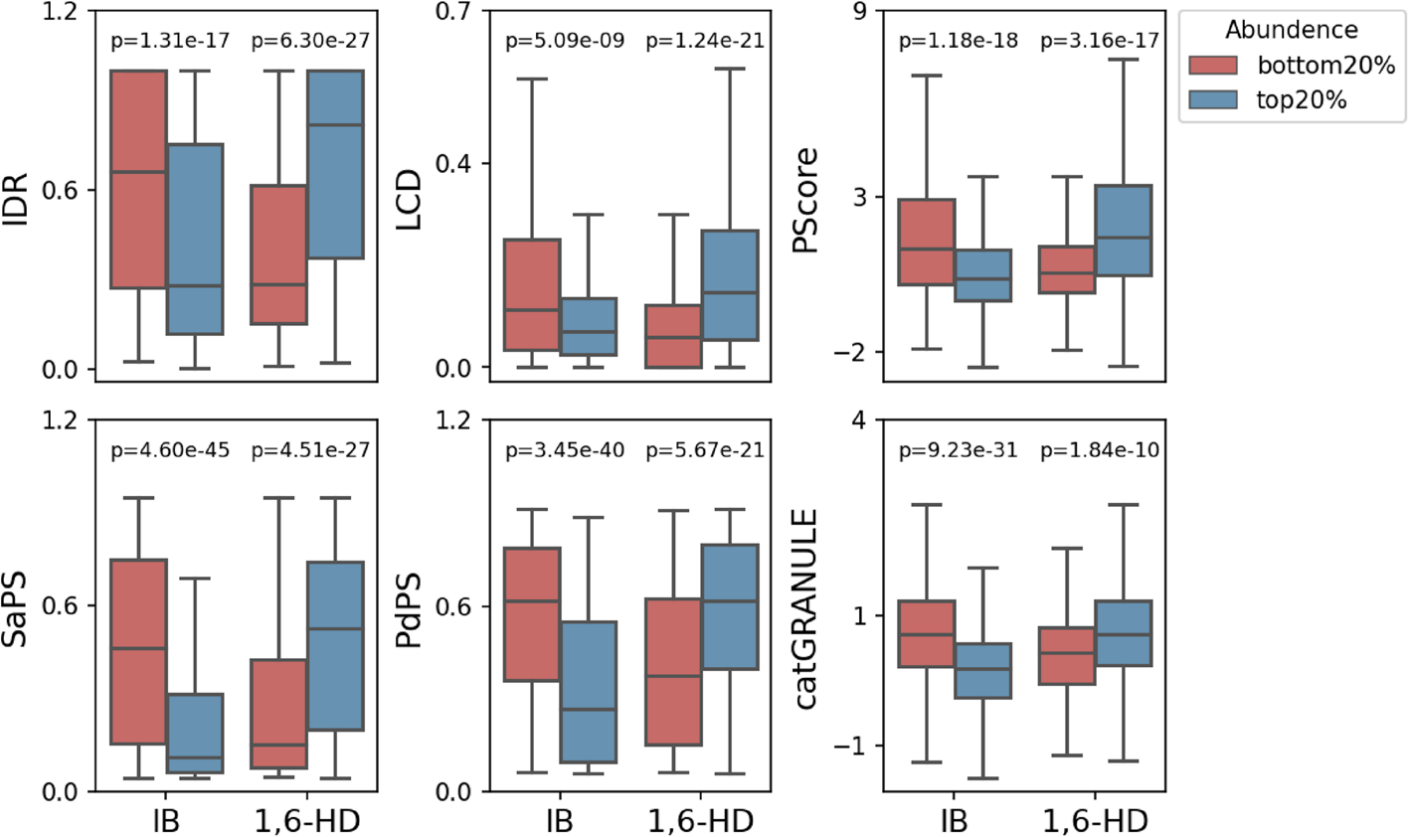
Analysis of known sequence features related to phase separation and phase separation prediction scores. IDR and LCD scores are their respective ratios to the full length of the protein. *P*-value was calculated using Mann–Whitney rank sum test. IDR, intrinsically disorder region; LCD, low complexity domain

Previous studies have revealed a number of LLPS-related sequence features [1, 19–21], most of which involve multivalent interactions. In brief, multivalent interactions could be mediated by intrinsically disordered regions (IDRs) or low complexity domains (LCDs). Therefore, we analysed the sequence characteristics of the enriched proteins in different treatment groups. We found that the high-abundance proteins in the 1,6-HD treatment group contained a high proportion of IDR or LCD regions compared with the low abundance proteins, whereas the trend in the IB treatment group was opposite (Fig. 3), indicating that 1,6-HD treatment could specifically enrich proteins with high proportion of disordered regions. In addition, many computational approaches have been developed to predict protein’s probability to undergo LLPS [22]. PScore was developed on the basis of pi–pi interaction frequency to screen LLPS proteins [23]. catGRANULE was initially trained to predict inappropriate liquid phase separation on the basis of yeast proteome [24]. PhaSePred provides self-assembling and partner-dependent phase-separating protein prediction [25]. Hence, to further explore the phase separation characteristics of the enriched proteins in the 1,6-HD treatment group, we compared the phase separation prediction scores of the enriched proteins in different groups (Fig. 3). The high-abundance proteins in the 1,6-HD treatment group exhibited higher phase separation scores in all four phase separation protein predictors (PScore, SaPS, PdPS, and catGRANULE) than the low-abundance proteins. By contrast, the enriched proteins in the IB treatment group showed lower phase separation scores in all four predictors. Among them, SaPS and PdPS predicted the likelihood of self-assembled phase separation proteins and interaction-dependent phase separation proteins, respectively, indicating that 1,6-HD treatment could enrich these two types of phase separation proteins well (Fig. 3). To further support our argument, we compared it with previously published gradient salt extraction experiments [26], and found proteins enriched in 1,6-HD treatment were prone to LLPS compared with salt treatment (Additional file 5: Figure S3).

These results suggest that 1,6-HD treatment could specifically disrupt the biomolecular condensates in the nucleus and thus enrich proteins prone to LLPS.

### Different gradients of 1,6-HD capture proteins with diverse physicochemical properties

**Fig. 4:**
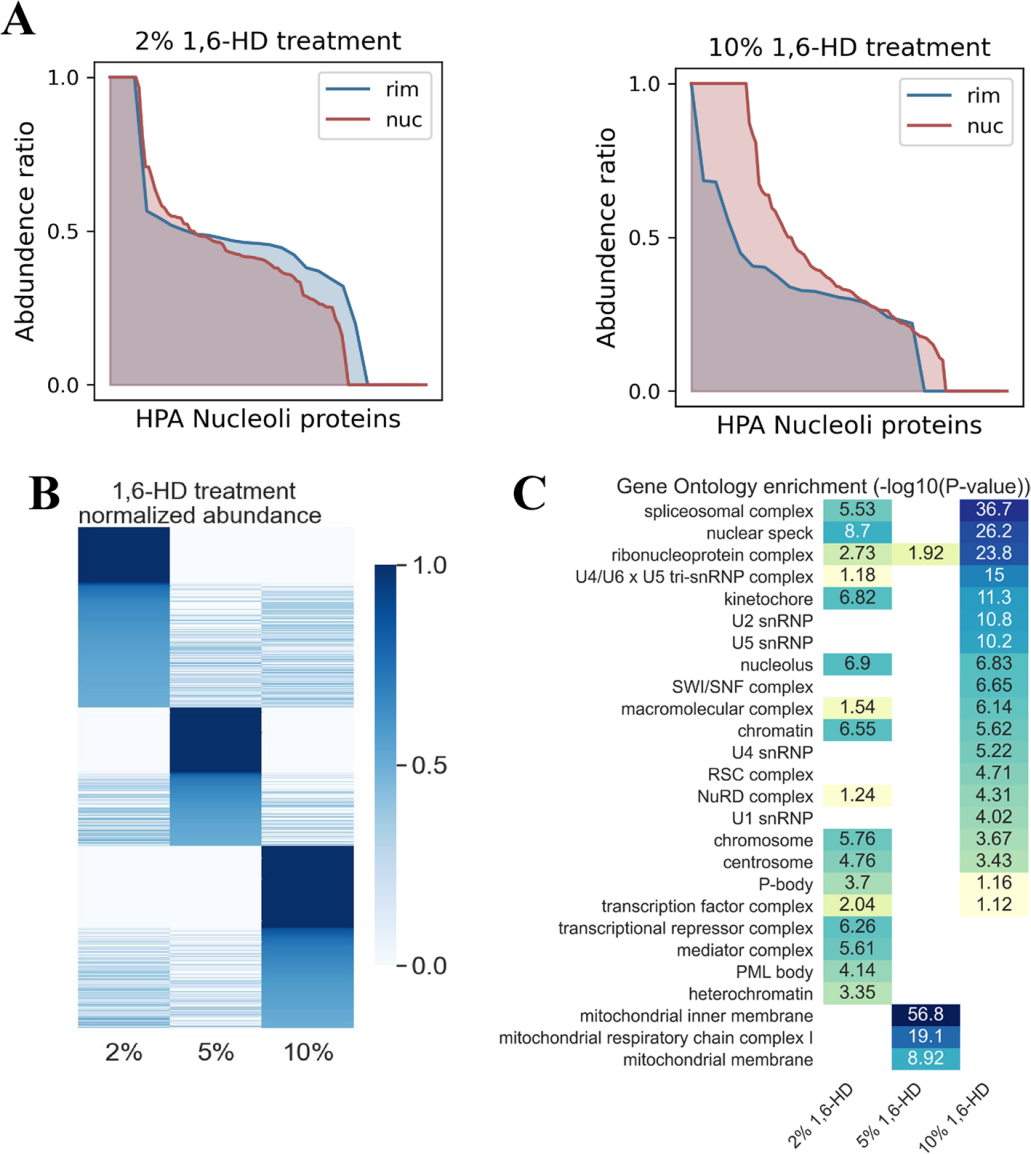
Representative proteins enriched by different gradients of 1,6-HD treatment. **A** Abundance ratio of nucleolar proteins in different sublocations. The x axis represents the nucleolus-associated proteins defined in HPA, and the y axis represents the ratio of protein abundance for a given 1,6-HD concentration treatment to the sum of abundance of all captured protein (abundance ratio). HPA, human protein atlas. **B** Ratio of protein abundance captured by gradient 1,6-HD elution to the total protein abundance captured by all concentration treatment groups. **C** Gene Ontology enrichment analysis of representative proteins. -Log10(*p*-value) was noted in the corresponding cell

To investigate the differences in protein sensitivity towards different gradients of 1,6-HD, we analysed the abundance and physiochemical properties of enriched proteins eluted by different concentrations of 1,6-HD. Because the nucleolus consists of multiple layers in its structure, we speculate that different concentrations of 1,6-HD have different influences on each layer. We classified nucleolus rim proteins (rim) and nucleolus interior proteins (nuc) with subcellular localization information provided by HPA. Then, we analysed their enrichment in treatment groups with different 1,6-HD concentrations. We found that a lower concentration of 1,6-HD (2%) enriched higher abundance of rim proteins while a higher concentration (10%) enriched higher abundance of nuc proteins (Fig. 4A). This indicates that the disruption ability of 1,6-HD on biomolecular condensates gradually increased with the increase of 1,6-HD concentration, which also further verified the effectiveness of different 1,6-HD concentration treatments in CHS-MS experiments.

To further explore the characteristics of the enriched proteins in different 1,6-HD treatment groups, we defined the proteins captured in each concentration treatment group with abundance exceeding 50% of the total abundance of all concentration treatment groups as representative proteins for this concentration (Fig. 4B, Additional file 6: Figure S4). We performed GO enrichment analysis on all representative proteins, and found that 2% 1,6-HD treatment group enriched transcription-related complex (transcription factor complex and mediator complex), and 10% 1,6-HD treatment group enriched splicing-associated complex and chromatin remodeling complex (nuclear speckle, spliceosomal complex, and SWI/SNF complex), while 5% 1,6-HD treatment group enriched mitochondrion-associated complex (mitochondrial inner membrane and mitochondrial respiratory chain complex I) (Fig. 4C). These results suggested that transcription-related complexes were sensitive to 2% 1,6-HD treatment, and splicing-related complexes were sensitive to 10% 1,6-HD treatment. In addition, we also defined the proteins with abundance share between 20 and 50% as common proteins (Additional file 7: Table S3) and found that the common proteins were mostly components of cytoplasmic localization (Additional file 8: Table S4).

**Fig. 5:**
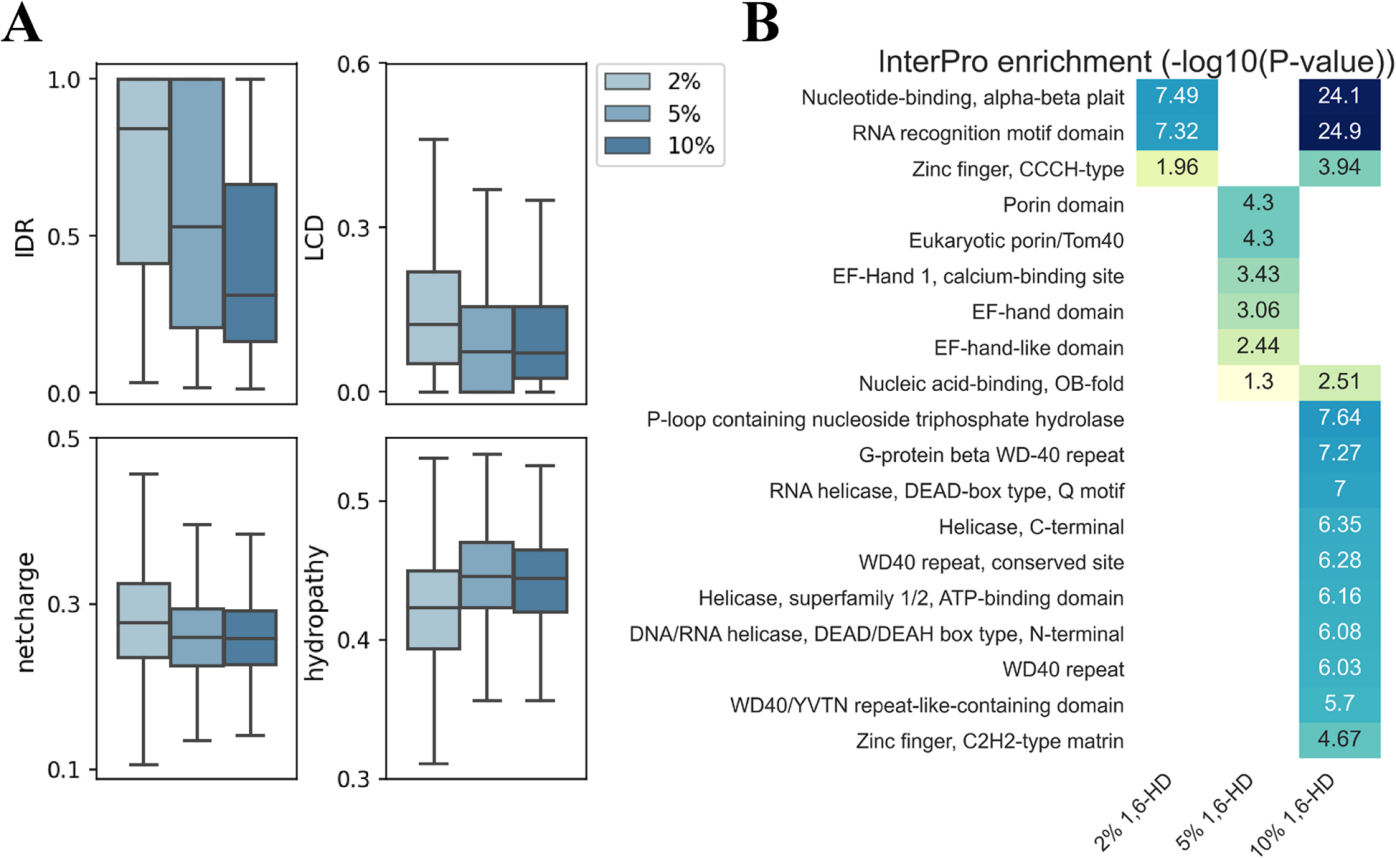
Analysis of physicochemical properties of representative proteins under different gradients of 1,6-HD treatment. **A** Distribution of physicochemical properties of representative proteins in different gradients of 1,6-HD treatment. **B** InterPro enrichment analysis of representative proteins. -Log10(*p*-value) was noted in the corresponding cell

We further analysed the sequence characteristics of all representative proteins, and found the representative proteins in the 2% 1,6-HD treatment group contained a higher proportion of IDR and LCD regions compared to the higher concentration treatment groups (Fig. 5A). These results indicate that the enriched proteins in the 2% 1,6-HD treatment group may rely mainly on IDR and LCD to maintain hydrophobic interactions with other proteins and nucleic acids, and are most sensitive to the 1,6-HD treatment. In addition, we found that the ratio of hydrophobic amino acids and charged amino acids also differed between the representative proteins in the different concentration treatment groups. Briefly, the representative proteins in the 2% 1,6-HD treatment group contain the lowest ratio of hydrophobic amino acids and the highest ratio of charged amino acids, suggesting that proteins containing fewer hydrophobic amino acids are more sensitive to 1,6-HD treatment (Fig. 5A). This result is consistent with previous findings [27–30] that 1,6-HD interferes with weak hydrophobic protein–protein or protein-RNA interactions that are required for these dynamic, liquid-like assemblies to form. We also performed InterPro enrichment analysis of all representative proteins, and found that 10% treatment group enriched more nucleotide-binding and structural domains (Nucleotide-binding alpha–beta plait, RNA recognition motif domain, and WD40 repeat) than 2% and 5% treatment groups (Fig. 5B, Additional file 9: Table S5).

In summary, CHS-MS captured representative proteins sensitive to different concentrations of 1,6-HD, and these proteins have different physicochemical properties.

## Discussion

Enriching CAPs has always been a heated research area. MS-based proteomics could be used to measure system-level protein dynamics and help collect CAPs in large scale, but capturing CAPs, especially those involved in nuclear condensates through LLPS, is challenging. Many chromatin proteins are expressed transiently at low levels or are difficult to extract from the nucleus [31–33]. Previous experimental approaches enriching CAPs are comprehensive and failed to detect most expressed TFs and TCs [34–39]. In the present research, we combined strict isotonic solution elution and gradient 1,6-HD treatment to partially solve this restriction. The 1,6-HD treatment enriched more TFs, TCs and RBPs than the IB treatment. These results indicated that our newly-developed method CHS-MS could effectively enrich CAPs, which helps further elucidate their LLPS properties.

Developing a systematic experimental methodology to identify and characterize biomolecular condensates and the LLPS properties of their components is crucial for further development of the LLPS field. In this study, CHS-MS combines quantitative proteomics and gradient 1,6-HD elution to investigate the LLPS properties of CAPs. CHS-MS is a simple and easy-to-operate method for rapid enrichment of CAPs prone to LLPS. And gradient 1,6-HD elution could assist in exploring the properties of proteins with different sensitivities towards different concentrations of 1,6-HD. We found that transcription-related complexes were sensitive to 2% 1,6-HD treatment and the representative proteins of 2% 1,6-HD treatment had the highest percentage of IDRs and LCDs. Previous studies have also shown TFs and TCs have high percentages of IDRs and can form transcription-related condensates. In addition, the splicing-related complexes were sensitive to 10% 1,6-HD treatment and the representative proteins of 10% 1,6-HD treatment had the lowest percentages of IDRs and LCDs and enriched more nucleotide-binding and structural domains. These results suggested that aside from IDR and LCD-dependent LLPS, LLPS proteins may have other modes of interactions to form biomolecular condensates. Thus, our method provides a new perspective to explain the different sequence features of LLPS proteins and identify possible physicochemical properties of LLPS proteins.

Nevertheless, our method still has a few limitations, with one of them concerning histone modifications. Histone modification also plays critical roles in chromatin-associated condensates [40, 41]. We found 1,6-HD treatment group enriched greater abundance of histone acetylation-associated proteins than IB treatment group (Additional file 10: Figure S5, Additional file 11: Table S6). Given that histones bind tightly to DNA, the influences histone modifications have in nuclear condensates and their biological functions are difficult to explore with CHS-MS. Future studies should consider targeting specific biomolecular condensates by histone modifications with immunoprecipitation and thus determine the key factors driving LLPS in each class of condensates. In addition, we noticed that CHS-MS enriched mitochondria-associated proteins (Figs. 1C and 4C) while salt extraction experiment [26] also enriched these proteins (Additional file 12: Figure S6A). We further compared the abundance of mitochondria-associated proteins in whole-cell and CHS-MS extractions and found lower abundance of these proteins in CHS-MS extractions (Additional file 12: Figure S6B). Future researchers should improve nuclear extraction methods to effectively remove cytoplasmic components such as mitochondria. Another limitation of our study lies in the possible influence of RNAs in biomolecular condensate formation. By comparing the protein types of the captured proteins in the 1,6-HD and IB treatment groups, we found that 1,6-HD treatment captured more RBPs. This result suggested that RNA may also play an important role in nuclear condensates. A previous study has shown that non-coding RNAs (ncRNAs), which are spatially restricted molecules, form nuclear condensates via the process of “seeding” [42]. For example, nucleoli use ribosomal precursor RNAs to recruit protein aggregates [43, 44], and paraspeckles use long ncRNA nuclear enriched abundant transcript 1 (NEAT1) as the scaffold molecule to recruit other proteins for local aggregation [45]. The properties of ncRNAs allow them to contribute to the “seeding” of nuclear compartments. For instance, the transcription process generates multiple copies of ncRNAs, which accumulate in high concentrations near transcription sites. Moreover, these spatially restricted ncRNAs contain sequence motifs and secondary structures that could bind diffusible RNAs and proteins, allowing these diffusible molecules to accumulate at high concentrations locally. Therefore, further studies applying CHS-MS should consider capturing nuclear ncRNAs together with proteins, which could lead to an enhanced understanding of the formation of nuclear biomolecular condensates and the biological processes they participate in with the phase separation proteins and ncRNAs.

## Conclusions

In summary, the CHS-MS experimental method is more convenient and less time-consuming than the existing methods used to investigate the LLPS properties of CAPs. This method could also be extended to different biological systems to help researchers rapidly enrich representative proteins in different gradients of 1,6-HD and systematically explore their physicochemical properties.

## Materials and methods

### Cell culture

K562 (ATCC) cells were grown in RPMI 1640 (Gibco) and supplemented with 10% fetal bovine serum (Gibco) and streptomycin (Gibco) at 37 °C with 5% CO_2_.

### Protein extraction

To separate cytosolic and nuclear fractions, a Minute™ cytoplasmic and nuclear extraction kit (#SC-003; Invent Biotechnologies) was used according to the manufacturer’s protocol. Briefly, 10^6^ K562 cells were washed in cold PBS and lysed following incubation with 200 μl cytoplasmic extraction buffer on ice for 5 min with vigorous vortexing for 15 s. Next, the lysates were centrifuged at 14,000 × g for 5 min at 4 ℃ to obtain the cytosolic and membrane fractions (supernatant) and nuclear fraction (pellet). The nuclear pellet was then washed with 0.5 ml cold PBS and centrifuged at 8,000 × g for 5 min at 4 ℃. Sequentially, 100 μl of cold PBS, 2%, 5%, and 10% 1,6-HD dissolved in PBS were added to the pellet by mixing, incubated for 5 min at 4 ℃, and centrifuged at 14,000 × g for 30 s at 4 ℃ to obtain the IB, 2%, 5%, and 10% 1,6-HD fractions.

### LC–MS/MS analysis

Samples were analysed on Orbitrap Fusion Lumos Plus mass spectrometers (Thermo Fisher Scientific, Rockford, IL, USA) coupled with an Easy-nLC 1000 nanoflow LC system (Thermo Fisher Scientific). Dried peptide samples were re-dissolved in solvent A (0.1% formic acid in water), loaded to a trap column (100 μm × 2 cm, homemade; particle size, 3 μm; pore size, 120 Å; SunChrom, USA) with a max pressure of 280 bar by using solvent A, and then separated on a homemade 150 μm × 12 cm silica microcolumn (particle size, 1.9 μm; pore size, 120 Å; SunChrom, USA) with a gradient of 5%–35% mobile phase B (acetonitrile and 0.1% formic acid) at a flow rate of 600 nl/minutes for 75 min. For detection with Fusion Lumos MS, a precursor scan was carried out in the Orbitrap by scanning m/z 300 − 1400 with a resolution of 120,000 at 200 m/z. The most intense ions selected under top-speed mode were isolated in Quadrupole with a 1.6 m/z window and fragmented by higher-energy collisional dissociation with normalized collision energy of 35% and then measured in the linear ion trap by using the rapid-ion trap scan rate. The automatic gain control targets were 5 × 10^5^ ions with a max injection time of 50 ms for full scans and 5 × 10^3^ with 35 ms for MS/MS scans. The dynamic exclusion time was set as 18 s. Data were acquired using the Xcalibur software (Thermo Scientific).

### Peptide identification and protein quantification

Raw sequencing data were searched against the National Center for Biotechnology Information Ref-seq human proteome database in Firmiana implemented with the Mascot search engine (Matrix Science, version 2.3.01) [46]. The mass tolerances were set as 20 ppm for precursor ions and 0.05 Da for product ions; N-acetylation and oxidation of methionine were set as variable modifications; and cysteine carbamidomethylation was set as a fixed modification. The peptide FDR was 1%. Proteins with at least one unique peptide and two strict peptides or more than two strict peptides (mascot ion score  ≥ 20) were defined as high-confidence proteins. The high-confidence proteins detected in at least one sample were selected for subsequent analysis to further increase the reliability. Peak area values were used to calculate protein quantification. The missing data were inputted with the minimum values. Quantile normalization was applied after missing value imputation.

### Statistical analysis

*P*-value was calculated to measure the statistical significance of TF, TC and RBP enriched in 1,6-HD treatment group by chi-square test. *P*-value was calculated to measure the statistical significance of protein abundance difference of different treatment groups by independent samples t test and Mann–Whitney rank sum test.

### Gene ontology enrichment analysis

Gene ontology analysis was conducted using Enrichr [47].

### Protein annotations

The TF and coactivator annotations are from animalTFDB [48]. The RNA binding protein annotations are from EuRBPDB [49]. The nuclear puncta protein annotations are from OpenCell [17]. The nucleolar rim and nuc protein annotations are from HPA [50], where the nuc portion of the protein includes nucleoli and nucleolus fibrillar center, as defined in HPA. Mitochondria-associated protein and histone-related protein annotations are from Gene Ontology [51].

### Protein sequence analysis and LLPS annotations

IDRs were predicted using ESpritz [52], with a threshold set at 5% FPR. The LCDs were predicted using SEG [53] under default parameters. The scores shown in the present paper are the ratio of IDRs and LCDs to the full length of the protein sequence. PScore [23] and catGRANULE [24] scorings were calculated under default parameters. SaPS and PdPS scorings were provided by PhaSePred [25] for the eight features scoring. Charged amino acid proportions were calculated by localCIDER [54] using get_FCR, and hydropathy score was calculated using get_uversky_hydropathy.

### Supplementary Information

**Additional file 1:** **Figure S1. **Protein detection in CHS-MS.

**Additional file 2:** **Table S1. **Proteins captured by CHS-MS.

**Additional file 3:** **Table S2. **List of matched GO terms of all proteins captured by IB and 1,6-HD treatment.

**Additional file 4:** **Figure S2. **Biomolecular condensates in CHS-MS.

**Additional file 5:** **Figure S3. **Analysis of salt extraction experiment versus CHS-MS.

**Additional file 6:** **Figure S4. **Representative proteins of different gradients of 1,6-HD treatment.

**Additional file 7:** **Table S3.** Representative proteins captured by different gradients of 1,6-HD treatment.

**Additional file 8:** **Table S4. **List of matched GO terms of all representative proteins.

**Additional file 9:** **Table S5. **List of matched InterPro domains of all representative proteins.

**Additional file 10:** **Figure S5. **Proteins related to histone in CHS-MS.

**Additional file 11:** **Table S6. **Proteins related to histone in CHS-MS.

**Additional file 12:** **Figure S6. **Mitochondria-related proteins in CHS-MS.

## Data Availability

All study data are included in the article and supporting information.

## Abbreviations

1,6-HD: 1,6-Hexanedial
AICAP: Anti-1,6-HD index of chromatin-associated proteins
CAPs: Chromatin-associated proteins
CHS-MS: Chromatin enriching hexanediol separation coupled with liquid chromatography-mass spectrometry
HP1α: Heterochromatin protein 1α
IDRs: Intrinsically disordered regions
IB: Isotonic buffer
LCDs: Low complexity domains
LLPS: Liquid-liquid phase separation
MS: Mass spectrometry
ncRNAs: Non-coding RNAs
NEAT1: Nuclear enriched abundant transcript 1
RBPs: RNA-binding proteins
TCs: Transcriptional cofactors
TFs: Transcription factors
