# Supplementary material for: Investigating phase separation properties of chromatin-associated proteins using gradient elution of 1,6-hexanediol
Source: BMC Genomics. 2023 Aug 28;24:493. doi: 10.1186/s12864-023-09600-1 (PMC10464338; doi:10.1186/s12864-023-09600-1)
Supplement: Supplementary file 1 — Additional file 1: Figure S1. Protein detection in CHS-MS. [file 12864_2023_9600_MOESM1_ESM.pdf]

Figure S1

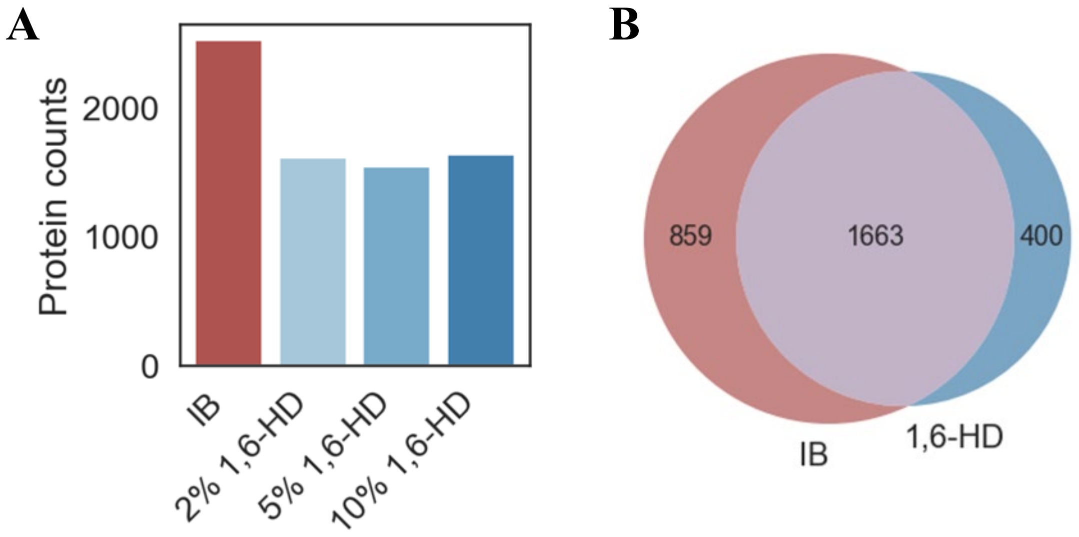

Figure S1. Protein detection in CHS-MS.

A Number of proteins detected in CHS-MS. **B** Venn diagram showing the overlap of proteins between IB and 1,6-HD treatment groups.
