# Supplementary material for: Investigating phase separation properties of chromatin-associated proteins using gradient elution of 1,6-hexanediol
Source: BMC Genomics. 2023 Aug 28;24:493. doi: 10.1186/s12864-023-09600-1 (PMC10464338; doi:10.1186/s12864-023-09600-1)
Supplement: Supplementary file 5 — Additional file 5: Figure S3. Analysis of salt extraction experiment versus CHS-MS. [file 12864_2023_9600_MOESM5_ESM.pdf]

# Figure S3

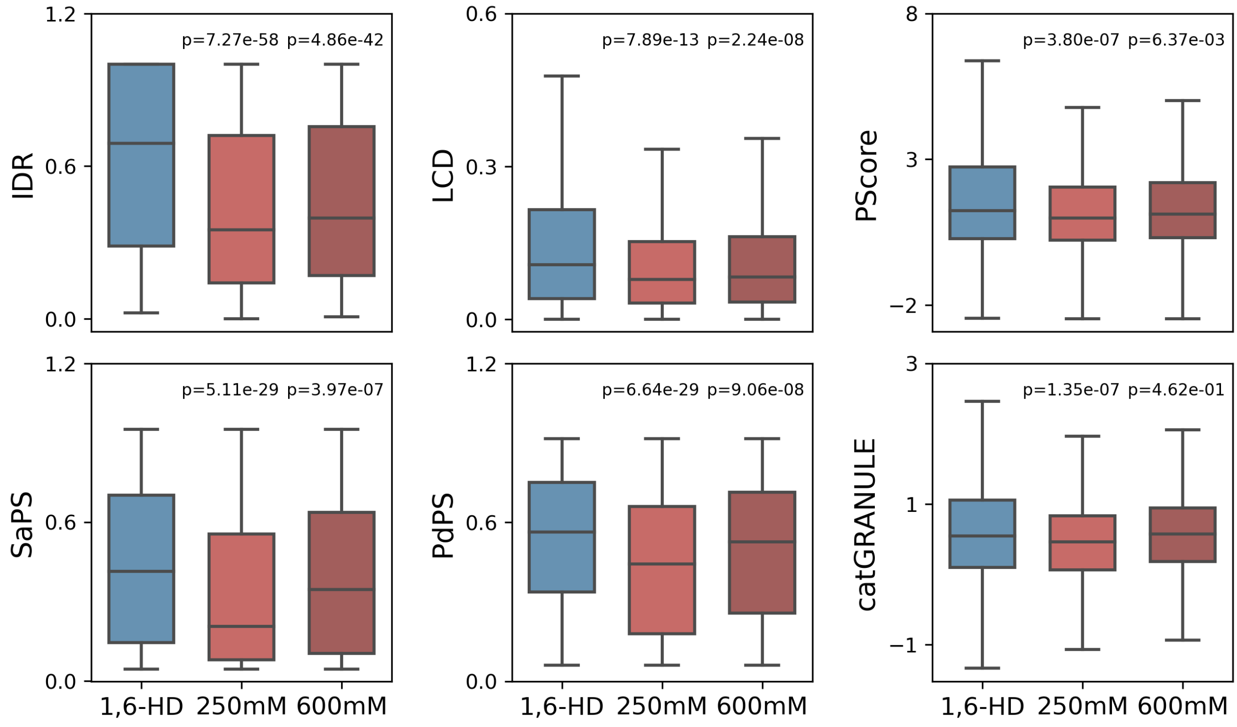


**Figure S3. Analysis of salt extraction experiment versus CHS-MS.**

IDR and LCD scores are their respective ratios to the full length of the protein. Different boxes indicated proteins with abundance exceeding 50% of the total abundance of all concentration treatment groups. *P*-value was calculated using Mann-Whitney rank sum test. IDR, intrinsically disorder region; LCD, low complexity domain.
