# Supplementary material for: Investigating phase separation properties of chromatin-associated proteins using gradient elution of 1,6-hexanediol
Source: BMC Genomics. 2023 Aug 28;24:493. doi: 10.1186/s12864-023-09600-1 (PMC10464338; doi:10.1186/s12864-023-09600-1)
Supplement: Supplementary file 6 — Additional file 6: Figure S4. Representative proteins of different gradients of 1,6-HD treatment. [file 12864_2023_9600_MOESM6_ESM.pdf]

Figure S4

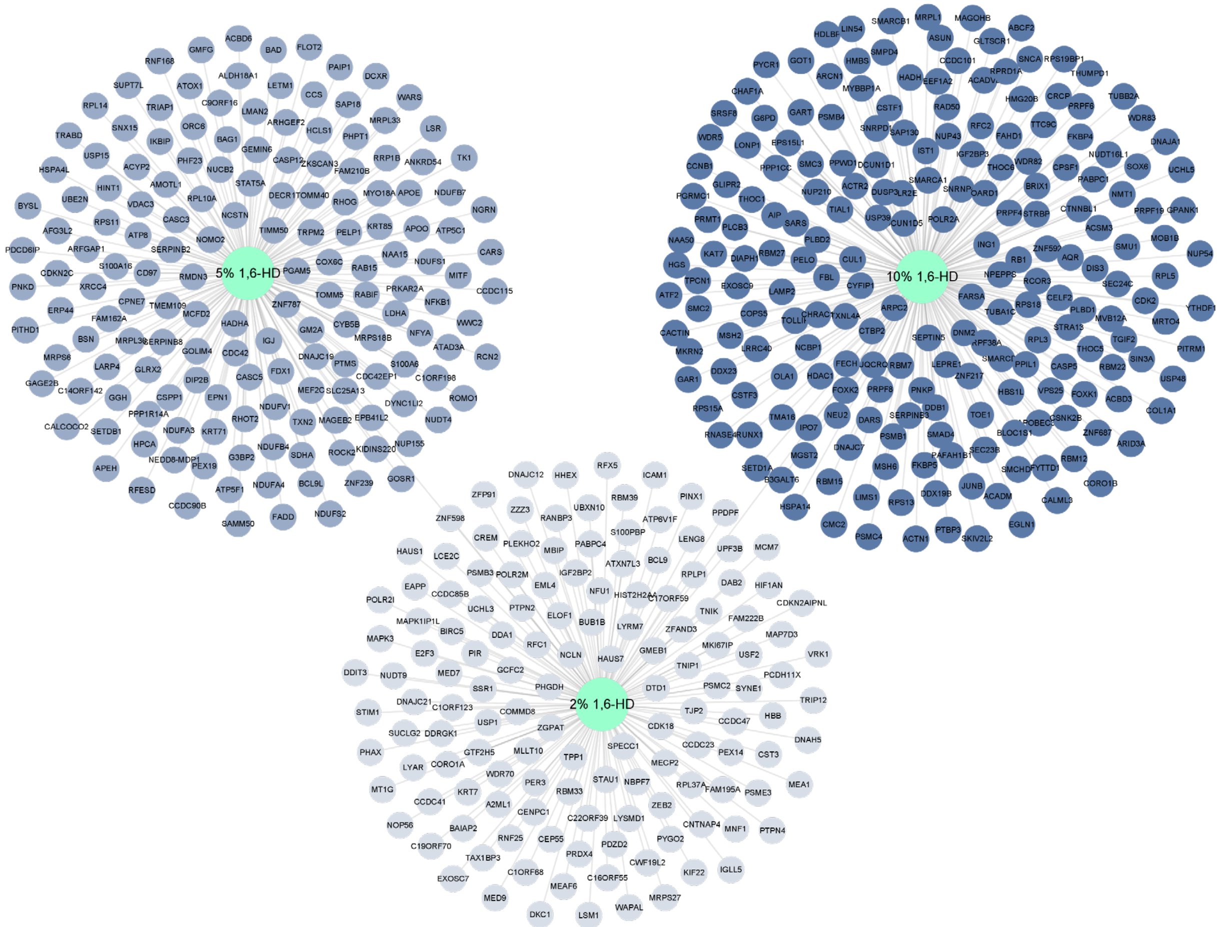

Figure S4. Representative proteins of different gradients of 1,6-HD treatment.

Representative proteins captured by gradient 1,6-HD elution with 100% abundance ratio.
