# Supplementary material for: Investigating phase separation properties of chromatin-associated proteins using gradient elution of 1,6-hexanediol
Source: BMC Genomics. 2023 Aug 28;24:493. doi: 10.1186/s12864-023-09600-1 (PMC10464338; doi:10.1186/s12864-023-09600-1)
Supplement: Supplementary file 10 — Additional file 10: Figure S5. Proteins related to histone in CHS-MS. [file 12864_2023_9600_MOESM10_ESM.pdf]

Figure S5

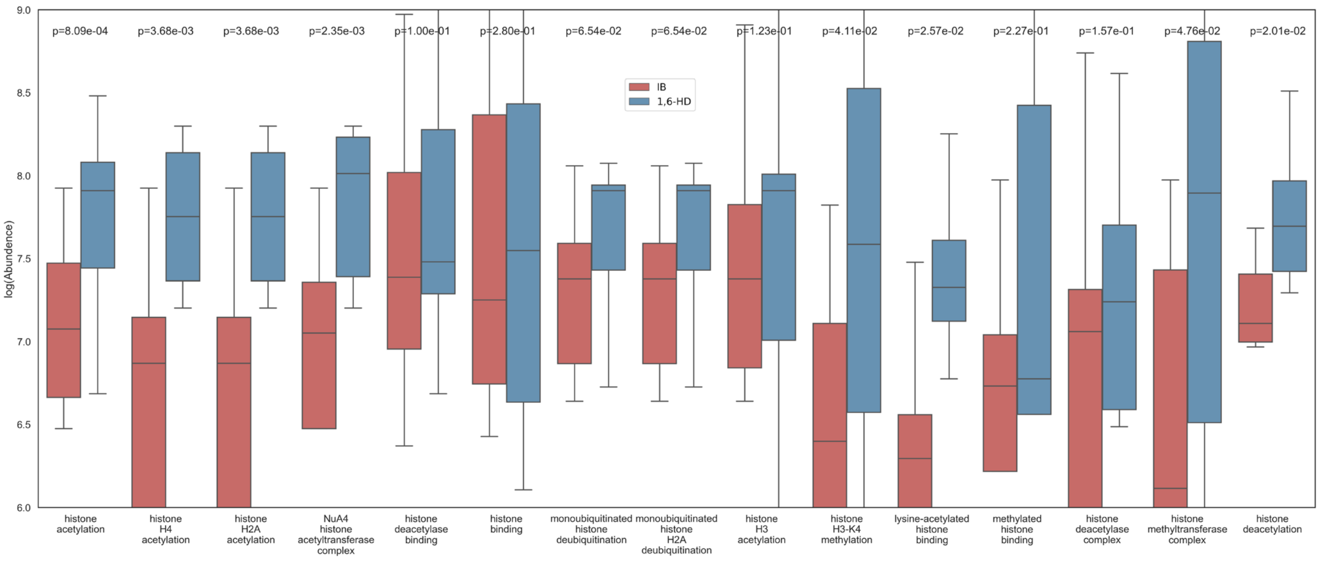

Figure S5. Proteins related to histone in CHS-MS.

Log10(abundance) of proteins related to histone. *P*-value was calculated using Mann-Whitney rank sum test. 1,6-HD, 1,6-hexanediol.
