# Supplementary material for: Investigating phase separation properties of chromatin-associated proteins using gradient elution of 1,6-hexanediol
Source: BMC Genomics. 2023 Aug 28;24:493. doi: 10.1186/s12864-023-09600-1 (PMC10464338; doi:10.1186/s12864-023-09600-1)
Supplement: Supplementary file 12 — Additional file 12: Figure S6. Mitochondria-related proteins in CHS-MS. [file 12864_2023_9600_MOESM12_ESM.pdf]

Figure S6

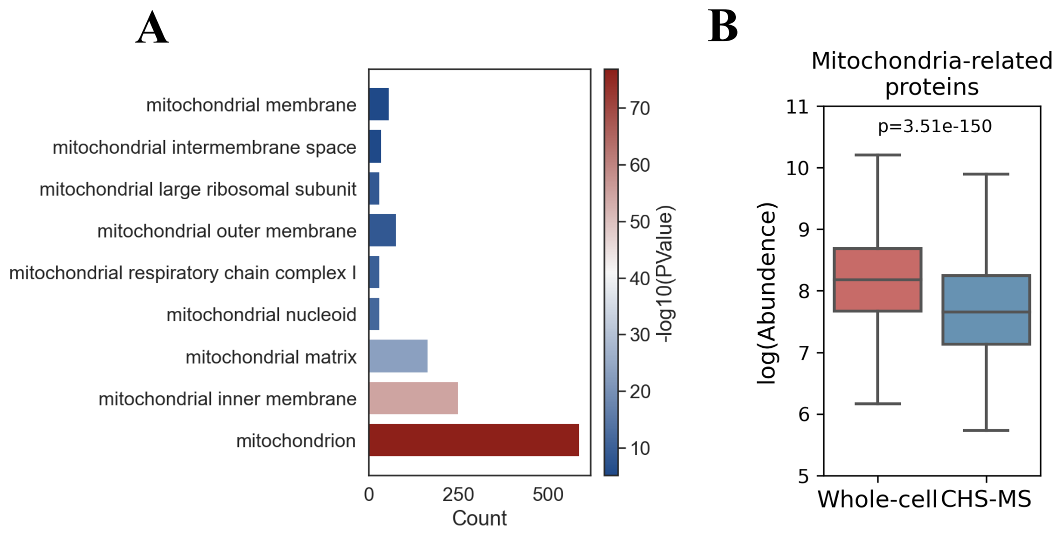

Figure S6. Mitochondria-related proteins in CHS-MS.

A Gene Ontology enrichment analysis of proteins captured by salt extraction experiment. **B** Comparison abundance of proteins related to mitochondria between whole-cell and CHS-MS extracts. *P*-value was calculated using Mann-Whitney rank sum test.
